# Characterization of a Novel *N*-Acylhomoserine Lactonase RmmL from *Ruegeria mobilis* YJ3

**DOI:** 10.3390/md16100370

**Published:** 2018-10-08

**Authors:** Xiulei Cai, Min Yu, Hu Shan, Xiaorong Tian, Yanfen Zheng, Chunxu Xue, Xiao-Hua Zhang

**Affiliations:** 1 College of Marine Life Sciences, Ocean University of China, Qingdao 266003, China; xlcai_99@163.com (X.C.); yumin@ouc.edu.cn (M.Y.); tianxr123@sina.com (X.T.); zhengyf90@126.com (Y.Z.); xuechunxu@outlook.com (C.X.); 2 College of Veterinary Medicine, Qingdao Agricultural University, Qingdao 266109, China; shanhu67@163.com; 3 Laboratory for Marine Ecology and Environmental Science, Qingdao National Laboratory for Marine Science and Technology, Qingdao 266071, China

**Keywords:** *N*-acylhomoserine lactonase, quorum quenching, *Ruegeria mobilis*, RmmL

## Abstract

Gram-negative bacteria utilize *N*-acylhomoserine lactones (AHLs) as quorum sensing (QS) signaling molecules for intercellular communication. Cell-to-cell communication depends on cell population density, and AHL-dependent QS is related to the production of multiple genes including virulence factors. Quorum quenching (QQ), signal inactivation by enzymatic degradation, is a potential strategy for attenuating QS regulated bacterial infections. Both Gram-positive and -negative bacteria have QQ enzymes that can degrade AHLs. In our previous study, strain *Ruegeria mobilis* YJ3, isolated from healthy shrimp, showed strong AHLs degradative activity. In the current study, an AHL lactonase (designated RmmL) was cloned and characterized from *Ruegeria mobilis* YJ3. Amino acid sequence analysis showed that RmmL has a conserved “HXHXDH” motif and clusters together with lactonase AidC that belongs to the metallo-β-lactamase superfamily. Recombinant RmmL could degrade either short- or long-chain AHLs in vitro. High-performance liquid chromatography analysis indicated that RmmL works as an AHL lactonase catalyzing AHL ring-opening by hydrolyzing lactones. Furthermore, RmmL can reduce the production of pyocyanin by *Pseudomonas aeruginosa* PAO1, while for the violacein and the extracellular protease activities by *Chromobacterium violaceum* CV026 and *Vibrio anguillarum* VIB72, no significant reduction was observed. This study suggests that RmmL might be used as a therapeutic agent in aquaculture.

## 1. Introduction

Quorum sensing (QS), also known as “auto-induction”, was originally found in *Vibrio fisheri* [1]. QS is a bacterial cell-to-cell communication system, in which bacteria regulate gene expression in the environment in accordance with cell density by using diffusible signal molecules. Therefore, by bacterial communication, QS can regulate gene expression and harmonize their behaviors including biofilm formation, bioluminescence, antibiotic production, and virulence factor secretion. Many different types of QS molecules were identified including *N*-acylhomoserine lactones (AHLs), autoinducer-2 (AI-2), 2-heptyl-3-hydroxy-4-quinoline (*Pseudomonas quinolone* signal, PQS), and 2-heptyl-4(1*H*)-hydroxyquinoline, HHQ) [2]. Among them, AHLs and AI-2 are the two most studied types.

**Figure 1:**
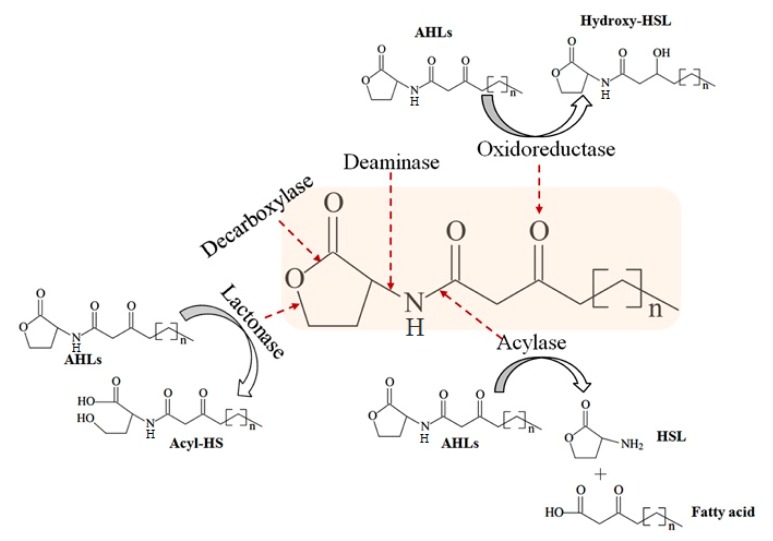
Enzymatic hydrolysis of *N*-acylhomoserine lactones (AHLs) [13].

By contrast, quorum quenching (QQ) can disrupt the QS communication system by enzymatic degradation of signal molecules. Currently, these enzymes might be sorted into three major categories based on the type of reaction catalyzed: AHL lactonases (lactone hydrolysis), AHL acylases (cleavage of amide bond), and AHL oxidoreductases (oxidoreduction) (Figure 1) [2]. Among the three types of QQ enzymes, AHL lactonase and AHL acylase have been extensively studied. The first AHL-degrading enzyme, AiiA, was identified in *Bacillus* sp. strain 240B1, is a member of the metallo-β-lactamase superfamily, and can attenuate the virulence of *Erwinia carotovora* [3]. AiiA shows a wide substrate specificity and there is a “HXHXDH~60aa~H” motif in its amino acid sequence. Heterologous expression of *aiiA* reduced the production of virulence factors. Since then, several AHL lactonases such as AhlD, AiiM, AidC, and MomL, were found in both Gram-positive (*Arthrobacter* and *Microbacterium*) and -negative (*Chryseobacterium* and *Muricauda*) bacteria, respectively [4,5,6,7]. AiiD was the first identified AHL acylase from *Ralstonia* sp. XJ12B [8] in 2003. Subsequently, several AHL acylases were identified from different bacteria including *Anabaena* (AiiC) [9], *Streptomyces* (ahlM) [10], *Pseudomonas* (pvdQ) [11], and *Shewanella* (Aac) [12]. In addition, expression of Aac from the intestinal *Shewanella* in fish pathogenic *Vibrio anguilarum*, contributed to the reduction of AHL production and biofilm formation [12]. Therefore, QQ could serve as a promising strategy to control bacterial virulence.

*Ruegeria mobilis* is a Gram-negative marine bacterium belonging to the clade *Roseobacter*. It was reported that *R. mobilis* was a globally distributed marine bacterium [14], could produce antibacterial compound tropodithietic acid (TDA), and thus could be considered as a potential probiotic bacterium [15,16]. It remains to be determined whether QS and QQ are present in *R. mobilis*.

*R. mobilis* YJ3 was isolated from healthy shrimp larvae and confirmed to show excellent antibacterial activities against many fish pathogens, such as *Vibrio* spp., *Edwardsiella tarda*, and *Aeromonas hydrophila*. Furthermore, the ICPs and ECPs of YJ3 showed quorum quenching activity, and it disappeared when treated by heat or protease K (unpublished data). Here, based on the genome sequence of YJ3, a novel AHL-lactonase gene *RmmL*, from *R. mobilis* YJ3 was identified and cloned. Further characterization of enzymatic activity and the effects of RmmL on in vitro virulence factor production were performed.

## 2. Results

### 2.1. Identification of the Extracelluar QQ Enzyme in YJ3

**Figure 2:**
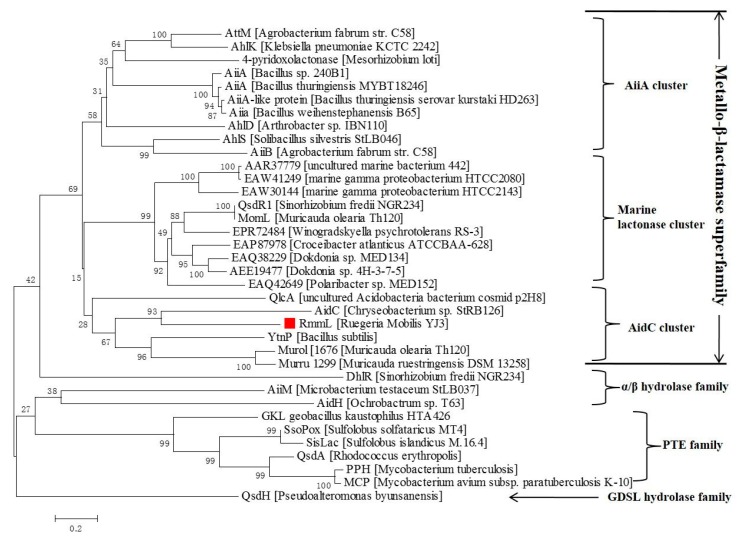
Neighbor-joining tree of AHL lactonases based on amino acid sequences. The sequences were from the AHL lactonases experimentally identified and unidentified (labeled with GenBank accession numbers). The dendrogram was constructed by the neighbor joining method with the MUSCLE program in the MEGA 7.0 software package (1000 bootstrap replicates). Bootstrap coefficients below 50% were not shown. Scale bar, 0.2 substitutions per amino acid position.

**Figure 3:**
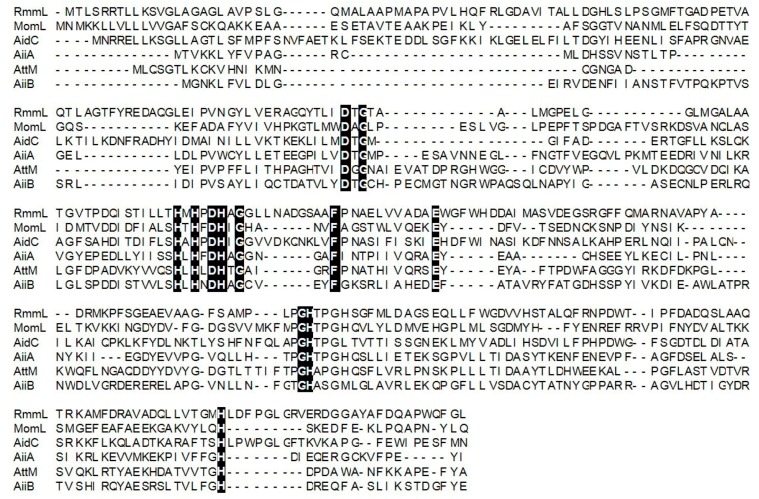
Multiple-sequence alignment of amino acid sequences of *Ruegeria Mobilis* Marine Lactonase (RmmL) and other representative AHL lactonases (MomL, AidC, AiiA, AttM, and AiiB). Sequence alignment was performed by the MUSCLE program in the MEGA 7.0 software. RmmL harbors an N-terminal signal peptide and the predicted cleavage site was marked by arrow. RmmL shares the “HXHXDH” motif with other AHL lactonases. The conserved sites are marked by a black background.

Local BLASTP (Basic Local Alignment Search Tool Program) of the annotated genome of *R. mobilis* YJ3, against a known QQ enzymes database, revealed that a protein, (L131) with 322 amino acid residues, is closely related to the known AHL lactonases. There was only 23–35% of amino-acid sequence identity shared with the known QQ enzymes (AiiA, AiiB, AttM, AhlS, AidC, AhlD) [3,4,7,17,18,19]. L131 could represent a novel marine-derived AHL lactonase, which was termed, RmmL (***R****uegeria* ***M****obilis* **M**arine **L**actonase). Phylogenetic analysis of RmmL and the other AHL lactonases from various bacteria showed that RmmL closely correlates to AidC under the AidC cluster previously reported (Figure 2) [2]. RmmL was predicted to be extracelluar with an N-terminal signal peptide of 30 amino acid residues through SignalP analysis [20]. Further sequence comparison analysis showed that RmmL has a “HXHXDH” zinc-binding motif conserved across different types of AHL lactonases (Figure 3).

### 2.2. Expression, Purification and Activity Test of RmmL in YJ3

**Figure 4:**
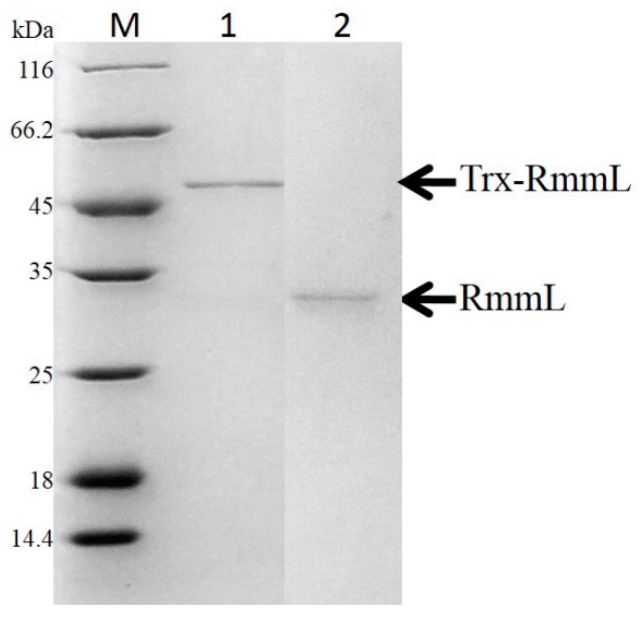
SDS-PAGE analysis of the affinity column-purified Trx-RmmL (50 kDa, lane 1) and RmmL protein (33 kDa, lane 2) after Trx-tag removal by enterokinase.

**Figure 5:**
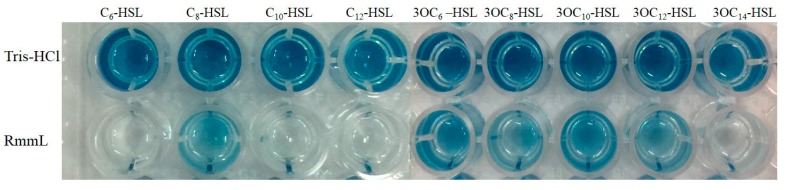
Characterization of the AHL degradative activity of RmmL by the A136 liquid X-Gal assay. Tris-HCl was used as the negative control. Signal molecules used are C_6_-HSL, C_8_-HSL, C_10_-HSL, C_12_-HSL, 3OC_6_-HSL, 3OC_8_-HSL, 3OC_10_-HSL, 3OC_12_-HSL, 3OC_14_-HSL.

The recombinant RmmL was then cloned into PET-32a and expressed in *E. coli* BL21 (DE3). The majority of the recombinant RmmL was expressed in a soluble form. The purified Trx-tag fusion protein Trx-RmmL and RmmL without Trx-tag produced a single band of 50 kDa and 33 kDa in size, respectively, which was matched with the predicted molecular weight of RmmL (Figure 4). The purified RmmL can degrade both short- and long-chain AHLs, and C_6_-, C_10_-, C_12_-, 3-oxo-C_14_-HSL are more efficiently degraded than C_8_-, 3-oxo-C_6_-, 3-oxo-C_8_-, 3-oxo-C_10_-, 3-oxo-C_12_-HSL (Figure 5), which proved that recombinant RmmL has AHL degradation activity.

### 2.3. RmmL is an AHL Lactonase

**Figure 6:**
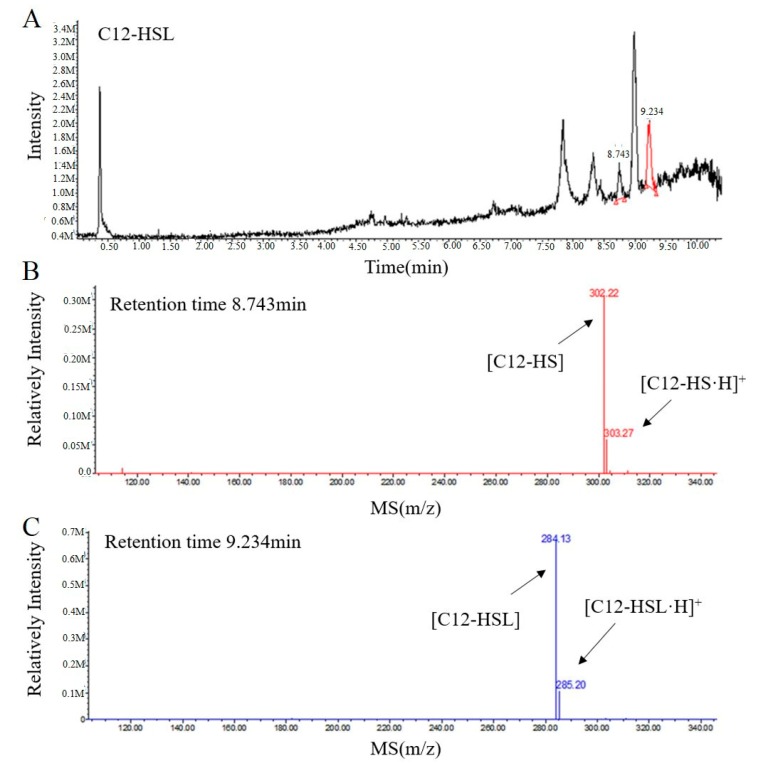
UPLC-MS analysis of the RmmL-hydrolyzed C_12_-HSL product. (**A**) UPLC profile of the RmmL-hydrolyzed C_12_-HSL product. ESI-MS analysis of UPLC fractions containing the 8.743-min product (**B**) and 9.234-min undigested C_12_-HSL (**C**).

To further elucidate the mechanism of RmmL catalysis, the resulting degradation products of C_12_-HSL, treated with purified RmmL, were analyzed by UPLC-MS. One major product with a retention time of 8.743 min was produced by the enzymatic digestion of C_12_-HSL. The catalytic product of RmmL reaction with C_12_-HSL gave a peak *m*/*z* of 302.22 with a mass increase of 18.09 in comparison with the *m*/*z* of intact C_12_-HSL (284.13) with a retention time of 9.234 min (Figure 6). The increased mass from C_12_-HSL to C_12_-HS resulted from an addition of a water molecule to the ester bond of C_12_-HSL due to the hydrolytic cleavage of lactone ring. This result proved that RmmL functions as an AHL lactonase which can hydrolyze the lactone ring of C_12_-HSL (Figure 1).

### 2.4. Biochemical Characterization of RmmL

**Figure 7:**
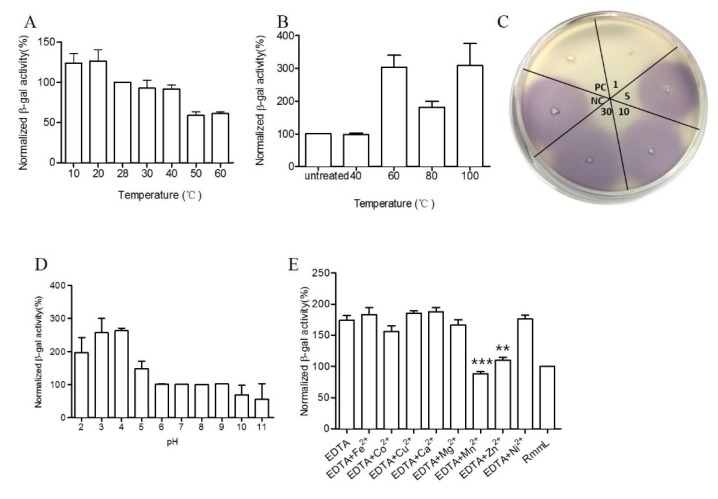
Biochemical characterization of RmmL activity. The optimal temperature (**A**), temperature stability at 40 °C, 60 °C, 80 °C, and 100 °C treatment for 30 min (**B**), and at 100 °C at 1 min, 5 min, 10 min 30 min (**C**), pH stability (**D**) of RmmL activity and the effect of metal ions and EDTA on RmmL activity (**E**) were measured. Bars indicate standard deviation of the mean. NC, negative control (Tris-HCl); PC, positive control (purified RmmL); Two-tailed unpaired *t*-test was used for statistical significance analysis; (**, *p* < 0.01; ***, *p* < 0.001; *n* ≥ 3).

RmmL exhibited high enzymatic activity at a relatively broad range of temperatures from 10 to 60 °C, with the optimal activity at 50 °C (Figure 7A). RmmL still retained 100% activity after heat treatment at 40 °C for 30 min but dropped at higher temperatures of 60 °C, 80 °C, and 100 °C (Figure 7B). In addition, the CV026 plate assay showed that RmmL still retained activity after incubation at 100 °C for 1 min, whereas it lost activity when treated at 100 °C for 5 min, 10 min, or 30 min (Figure 7C). Lower pH treatment at 4 °C for 3 h reduced the RmmL activity while higher pH treatment at 4 °C for 3 h retained or even increased enzymatic activity (Figure 7D). Either EDTA alone or together with various metal ions Ni^2+^, Mg^2+^, Ca^2+^, Cu^2+^, and Fe^2+^ partially inhibited RmmL activity, however, Zn^2+^, Mn^2+^, and Co^2+^ together with EDTA enhanced RmmL activity (Figure 7E). 

### 2.5. The Effect of RmmL on In Vitro Production of Virulence Factors 

**Figure 8:**
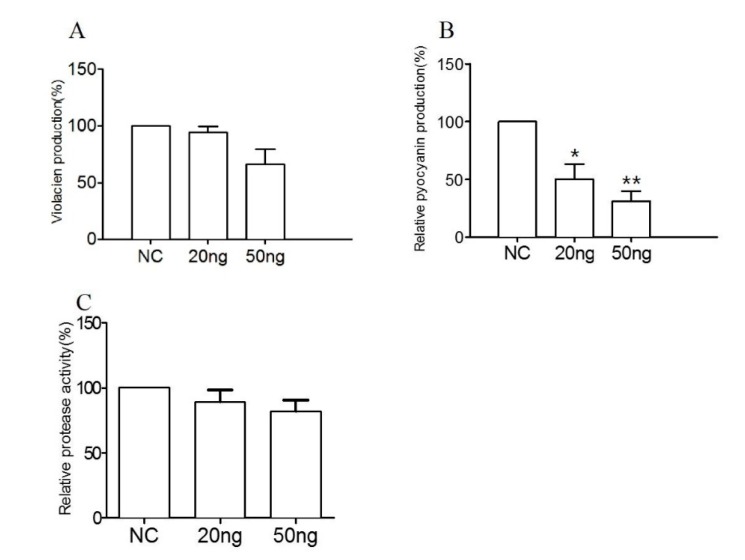
The effect of RmmL on virulence factor production. The violacein (**A**) and pyocyanin (**B**) production of *C. violaceum* and *P. aeruginosa*, respectively, and the extracellular protease activity (**C**) of *V. anguillarum* were determined. NC, negative control; Two-tailed unpaired t-test was used for statistical significance analysis; (*, *p* < 0.05; **, *p* < 0.01; n ≥ 3).

The effect of RmmL on violacien and pyocyanin production of *C. violaceum* VIR24 and *P. aeruginosa* PAO1, respectively, and the effect of RmmL on the extracellular proteolytic activity of *P. aeruginosa* PAO1 and *V. anguillarum* VIB72 were evaluated in vitro. RmmL reduced the violacien and pyocyanin production by *C. violaceum* VIR24 and *P. aeruginosa* PAO1, respectively, in a concentration–dependent response (Figure 8A,B). Similarly, RmmL reduced the extracellular protease activity of *V. anguillarum* VIB72 in a concentration–dependent manner (Figure 8C). Instead, the reduction of the extracellular protease activity of *P. aeruginosa* PAO1 was not detected, and the data was not shown.

## 3. Discussion

There are more than 30 types of AHL lactonases identified and reported. Phylogenetic analysis of amino acid sequences of all known lactonases has classified them into four different families including the metallo-β-lactamase superfamily, the phosphotriesterase (PTE) family, GDSL hydrolase family, and α/β hydrolase family [2]. Within the metallo-β-lactamase superfamily, three clusters of AiiA, marine lactonase, and AidC were further defined. In this study, a novel QQ enzyme of AHL lactonase, RmmL, from a marine *R. mobilis* YJ3 strain was identified and characterized. To our knowledge, this is the first study that has identified and characterized the QQ enzyme in *R. mobilis*. Phylogenetic tree analysis revealed that RmmL is distantly related to the marine lactonase cluster, and closely related to AidC derived from a terrestrial bacterium in the AidC cluster (Figure 2). It remains unclear if RmmL is an exception, or if there are two or more marine lactonase clusters. Several reports demonstrated that hydrolysis of the lactone ring by AHL lactonases results from the addition of a water molecule into AHLs to produce acylhomoserine [4,5,6,7], and in this research the UPLC-MS was used to confirm the AHL degradation activity of RmmL via a lactonase mechanism. The mass spectrum of the degraded product of C12-HSL treated with RmmL produced a correct peak corresponding to acylhomoserine at 302.22 *m*/*z*. This result proved that RmmL is an AHL lactonase.

RmmL can degrade both short- and long-chain AHLs, but is somewhat more effective at degrading C_6_-, C_10_-, C_12_-, 3-oxo-C_14_-HSL (Figure 5). The activity of degrading C_4_-HSL was not detected in this research. Further biochemical characterization demonstrated that RmmL has the same optimal temperature (50 °C) to AiiM derived from the leaf-associated bacterium *Microbacterium testaceum,* while other QQ enzymes, AidC, AdeH, and MomL, have lower optimum temperatures (30 °C, 35 °C, and 40 °C, respectively) [5,6,7,21]. Different from *M. testaceum*, which has a wide range of growth temperatures (10–60 °C), *R. mobilis* has a narrow range of growth temperatures, 5–35 °C. In addition, RmmL retained 100% activity after 30 min treatment at 40 °C and 1 min at 100 °C. These results suggest RmmL could tolerate a relatively high temperature and maintain better enzymatic activity. Similar to MomL, a low range of pH affects the stability of RmmL. This is likely due to the conventional chelating agent, citric acid, used in the buffer, as previously speculated [5].

RmmL might have a different AHL-degrading mechanism with other lactonase, even though the same “HXHXDH” motif, which agrees with the metallohydrolase criterion and conserved in the sequences of the metallo-β-lactamase superfamily, were found in RmmL as other AHL lactonases. EDTA (metal-chelating reagent) alone and most of the tested metal ions with EDTA inhibited the enzyme activity of RmmL, whereas Zn^2+^, Mn^2+^ and Co^2+^ with EDTA enhanced RmmL activity (Figure 6E), which is similar as AidC [7]. Despite the presence of “HXHXDH” zinc-binding motif in RmmL, its different metal-binding capability might result from the interaction involved with multiple sites, which remains to be determined by using site-directed mutagenesis in our future work. 

The bacterial pathogen *C. violaceum* uses a LuxIR-type quorum-sensing system and the QS molecule C_10_-HSL can regulate the production of the violacein [22]; While 3O-C_12_-HSL and C_4_-HSL, C_6_-HSL and 3O-C_6_-HSL, respectively, are the important AHL signal molecules of *P. aeruginosa* [23] and *V. anguillarum* [24]. RmmL significantly reduced the pyocyanin production of *P. aeruginosa*, while there was no significant reduction of the violacien production of *C. violaceum*, and extracellualar protease activity of *V. anguillarum* was observed. These results indicate that RmmL shows potential as a new therapeutic agent for attenuation of QS phenotypes in pathogenic bacteria. Future studies are warranted to test the therapeutic effect of RmmL on pathogens in vivo.

There are increasing numbers of studies identifying QS molecules and QQ enzymes from both Gram-positive and -negative bacteria of different families. In the case of the *Rhodobacteriaceae* family, only a few (*Phaeobacter inhibens*, *Ruegeria sp*. and *Dinoroseobacter shibae*) out of 17 different genera were reported to have produced AHL [25], but there is no report about identification of QQ enzymes from any genus of the *Rhodobacteriaceae* family except *R. mobilis* from this study. RmmL identified and characterized represents a novel AHL lactonase and might have the potential to be used as a therapeutic agent against fish pathogens in aquaculture.

## 4. Materials and Methods

### 4.1. Bacterial Strains, Media, Growth Conditions, and Chemicals

*R. mobilis* YJ3 was cultured in marine broth 2216 (MB; Becton Dickinson, Franklin Lakes, NJ, USA) at 28 °C. *Escherichia coli* strain BL21 (DE3) was cultured on Luria–Bertani (LB) agar at 37 °C and used as a host for expressing proteins whose encoding genes were cloned into pET-32a (Novagen, Madison, WI, USA). The AHL biosensor *Agrobacterium tumefaciens* A136 (pCF218) (pCF372) [26] was maintained on LB agar and grown in AT minimal medium (KH_2_PO_4_, 10.7 g/liter; MgSO_4_·7H_2_O, 160 mg/liter; CaCl_2_·2H_2_O, 10 mg/liter; FeSO_4_·7H_2_O, 5 mg/liter; MnSO_4_·7H_2_O, 2 mg/liter; (NH_4_)_2_SO_4_, 2 g/liter; glucose, 2 g/liter) [27] containing 0.5% (*w*/*v*) glucose for detecting AHLs in liquid X-Gal assay. The AHL biosensor *Chromobacterium violaceum* CV026 [28] and VIR24 [29], which were used to detect short-chain (C_4_ to C_8_) and long-chain (C_8_ to C_14_) AHLs, were maintained on LB agar at 28 °C. *Pseudomonas* *aeruginosa* PAO1 was cultured on tryptic soy agar (TSA, Oxoid, Lenexa, KS, USA) at 37 °C. Appropriate antibiotics were added at the following concentrations: Ampicillin, 100 µg mL^−1^; Spectinomycin, 50 µg mL^−1^; Tetracycline, 4.5 µg mL^−1^. C_4_-HSL, C_6_-HSL, 3-oxo-C_6_-HSL, and C_8_-HSL were purchased from Cayman Chemical Company (Ann Arbor, MI, USA); 3-oxo-C_8_-HSL, C_10_-HSL, 3-oxo-C_10_-HSL, C_12_-HSL, 3-oxo-C_12_-HSL, C1_4_-HSL, and 3-oxo-C_14_-HSL were purchased from Sigma-Aldrich (St. Louis, MO, USA). All of the AHL stock solutions (10 to 500 mM) were prepared in dimethyl sulfoxide (DMSO) and stored under −20 °C.

### 4.2. Cloning, Expression, and Purification of QQ Enzyme of YJ3

The putative lactonase gene *rmmL* of *R. mobilis* YJ3 was predicted from the whole genome sequence of YJ3 (X. Cai and X-H Zhang, unpublished data) by means of local BLASTP against several known QQ enzymes sequences. Genomic DNA of *R. mobilis* YJ3 was extracted through a phenol-chloroform method and used as the template of PCR (polymerase chain reaction) amplification for the *rmmL* gene using a pair of primers: Lactonase131F: 5′-CGCGGATCCATGACCCTTTCCCGT-3′ and Lactonase131R: 5′-CCCAAGCTTTTAGAGGCCGAATTGC-3′. The restriction sites for *Bam*HI and *Hind*III are underlined both in the forward and reverse primers, respectively. PCR was performed with the following conditions: 1 cycle of 98 °C for 30 s; 30 cycles of 98 °C for 10 s, 55 °C for 10 s, and 72 °C for 1 min; and a final extension step at 72 °C for 2 min. The amplified DNA fragment, digested by *Bam*HI and *Hind*III, was cloned into pET-32a vector (Novagen) to get the plasmid pET-32a-*rmmL*, which was then transformed into *E. coli* BL21 (DE3). Protein expression and purification were performed as previously described [5]. Briefly, *E. coli* BL21 (DE3) was grown in LB at 37 °C until the OD_600_ of the culture was between 0.4 to 0.6. Following this, 0.1 mM IPTG (isopropyl-d-thiogalactopyranoside) was added into the culture to induce protein expression at 16 °C with moderate shaking overnight. Following sonication and centrifugation, the Trx-tag fusion protein was purified from the supernatant by NTA-Ni column (Qiagen, Hilden, Germany). For purification of the RmmL without Trx-tag sequence, the fusion protein was incubated with enterokinase at room temperature overnight followed by purification using NTA-Ni column. The purified proteins were analyzed by SDS-PAGE and stored at 20 °C with 25% glycerol.

### 4.3. Liquid Chromatography-Mass Spectrometry (LC-MS) Analysis of AHL Degradation Products by RmmL

To analyze the chemical structure of products formed by RmmL catalysis, 100 µM C12-HSL was subjected to degradation by 10 µg purified RmmL in 500 µL reaction volume containing 50 µL 1,4-piperazinediethanesulfonic acid (PIPES, 1 M, pH 6.7) buffer. The hydrolysis reaction was carried out at 28 °C for 30 h and the mixture was extracted with 0.1% ethyl acetate three times and analyzed by ultra-performance liquid chromatography (UPLC) using a SunFire C18 reversed-phase column (3.5 µm; 4.6 by 50 mm) with a mobile phase of acetonitrile-water (0.01% trifluoroacetic acid; a linear gradient [*v*/*v*] of acetonitrile from 5 to 100% over 1.0 min at a flow rate of 0.5 mL min^−1^). The electrospray ionization (ESI)-mass spectrometry (MS) was used to analyze the resulting fractions.

### 4.4. Detection of AHL-Degrading Activity of RmmL against Different Signaling Molecules

The *A. tumefaciens* A136 liquid X-gal assay was carried out to determine the AHL-degrading activity of RmmL according to Tang et al. [30]. The normalized β-galactosidase activity was used to characterize the remaining AHLs, which is exactly the opposite of the activity of the RmmL. Briefly, a mixture of 8.9 µL recombinant RmmL, 1 µL PIPES, and 0.1 µL of 10 mM signaling molecule (C_6_-, C_8_-, C_10_-, C_12_-, 3-oxo-C_6_-, 3-oxo-C_8_-, 3-oxo-C_10_-, 3-oxo-C_12_-, 3-oxo-C_14_-HSL) were incubated for 30 h at 28 °C and then mixed with 190 µL A136 X-gal assay solution (*A. tumefaciens* A136 was cultured in broth overnight and then inoculated in AT minimal-glucose medium containing a final X-gal concentration of 250 µg mL^−1^) in 96-well plate and incubated at 28 °C for 24 h, The normalized β-galactosidase activities were calculated as described by Tang et al. [5]. 

### 4.5. Physical and Chemical Parameters That Affect RmmL Activity

The thermostability and pH stability of RmmL were measured by the A136 liquid X-gal assay as previously described. Purified RmmL were pre-treated at different temperatures (40 °C, 60 °C, 80 °C, and 100 °C) for 30 min and at different pHs (Na_2_HPO_4_/citric acid for pH 2.0 to 7.0, Tris-HCl for pH 8.0 to 9.0, and Na_2_CO_3_/NaHCO_3_ for pH 10.0 to 11.0) at 4 °C for 3 h, respectively, and the activity was measured by the A136 liquid X-gal assay method by incubating RmmL with 10 mM C_6_-HSL substrate in PIPES buffer. The enzymatic activity of RmmL at 28 °C or treated at pH 7.0 was set as 100%. In addition, to test the stability of RmmL at a high temperature, RmmL was treated at 100 °C for 1 min, 5 min, 10 min or 30 min, and then the CV026 plate assay method was used to detect the residual activity [24]. The optimal temperature of RmmL activity was measured by the A136 liquid X-gal assay method by incubating RmmL with 10 mM C_6_-HSL substrate in PIPES buffer at different temperatures (10 °C, 20 °C, 30 °C, 40 °C, 50 °C, and 60 °C). The activity of RmmL at 28 °C was used as a control for comparison. To evaluate the effect of metal ions and EDTA on RmmL, purified RmmL was first incubated with 1 mM EDTA only at 4 °C for 1 h, and then further treated with 1.1 mM of different metal ions (Ni^2+^, Mg^2+^, Mn^2+^, Ca^2+^, Cu^2+^, Zn^2+^, Co^2+^, and Fe^2+^) at 4 °C for 1 h, followed by measurement of the residue activity using the A136 liquid X-gal assay with 10 mM C_6_-HSL substrate in PIPES buffer.

### 4.6. The Effect of RmmL on Violacien and Pyocyanin Production

The biosensor *C. violaceum* VIR24 was grown in 5 mL LB broth containing different concentrations (20 ng and 50 ng) of purified RmmL protein for 24 h at 28 °C without shaking. The cell density of each culture was then measured at a wavelength of 660 nm. Two hundred microliters of each culture were used for extracting violacein as previously described [31]. The upper n-butyl alcohol phase was collected and used to measure the absorbance at a wavelength of 590 nm. The amount of violacien was calculated using the equation OD590/(OD660 × 200 µL). No RmmL was added in the negative control group. The effect of RmmL on the pyocyanin production of *P. aeruginosa* PAO1 was performed as described by Tang et al. [5].

### 4.7. The Effect of RmmL on the Extracellular Proteolytic Activity of V. anguillarum VIB72

*V. anguillarum* VIB72 (5 mL) was cultured together with different concentrations of RmmL (0, 25 ng, 50 ng), and then the extracellular proteolytic activity of the cultures was determined as described by Tang et al. [5].

### 4.8. Nucleotide Sequence Accession Number

The nucleotide sequence of *rmmL* gene from YJ3 has been deposited in the GenBank database under accession no. MH289473.
